# Nanosecond Electric Pulses Induce Early and Late Phases of DNA Damage and Cell Death in Cisplatin-Resistant Human Ovarian Cancer Cells

**DOI:** 10.1155/2018/4504895

**Published:** 2018-08-08

**Authors:** Guanhua Qian, Tinghe Yu

**Affiliations:** Key Medical Laboratory of Obstetrics and Gynecology, The Second Affiliated Hospital, Chongqing Medical University, Chongqing, China

## Abstract

Chemoresistance is a challenge for management of ovarian cancer, and therefore the response of resistant cells to nanosecond electric pulses (nsEP) was explored. Human ovarian cancer cell line COC1 and the cisplatin-resistant subline COC1/DDP were subjected to nsEP (32 ns, 10 kV/cm, 10 Hz pulse repletion frequency, and 10 min exposure duration), and then the cellular responses were followed. The percentages of dead cells and of comet-formed cells in the alkaline assay displayed two peak levels (i.e., 2 and 8 h after nsEP exposure), with the highest value noted at 8 h; the percentage of comet-formed cells in the neutral assay was increased at 8 h; the apoptotic percentage was increased at 8 h, with collapse of the mitochondrial membrane potential and the activation of caspase-3 and caspase-9. The comet assay demonstrated DNA single-strand break at 2 h and double-strand break at 8 h. nsEP resulted in lower cytotoxicity in COC1/DDP cells compared with COC1 cells. These findings indicated that nsEP induced early and late phases of DNA damage and cell death, and these two types of cell death may have distinct applications to treatments of chemoresistant ovarian cancers.

## 1. Introduction

Chemoresistance is yet a challenge for management of ovarian cancer; a chemical sensitizer lacks selectivity, resulting in poor therapeutic efficacy and toxicity to noncancerous tissues [1]. A physical modality may be an alternative because the energy can be delivered into the preselected volume without harming adjacent tissues, realizing a targeted treatment [2].

Nanosecond electric pulses (nsEP) can trans-membranously evoke a high potential (i.e., > 0.5–1.0 V, the critical potential required to cause damage) in specifically subcellular structures, thereby causing responses such as membrane poration, ion permeation, and protein modification [3–7]. These effects to a certain extent will lead to cell death mainly via inducing apoptosis [5–7]. However, the responsive difference between chemosensitive and chemoresistant cells remains unclear.

Theoretical calculations based on the multilayer dielectric model have manifested that nsEP (24 ns, 6 kV/cm) can evoke potentials of 1.98 V in the nucleoplasm, 1.17 V in the cytoplasm, and 0.25 V in the cellular membrane, in cisplatin-resistant human ovarian cancer cells COC1/DDP [4]. The high potential in the nucleoplasm leads to DNA single-strand break (SSB). The data suggest that nsEP may be a therapeutic strategy for resistant cancers, considering the pivotal role of DNA damage and repair in chemoresistance [8, 9]. However, the biological implications of the high potential in the cytoplasm remain unclear.

Here we compared the response to nsEP between cisplatin-sensitive and -resistant human ovarian cancer cells. Data indicated that nsEP can induce early and late phases of cell death in chemoresistant cells. These two types of cell death may have distinctly therapeutic applications.

## 2. Materials and Methods

### 2.1. Cells

Human ovarian cancer cell line COC1 and the cisplatin-resistant subline COC1/DDP (China Center for Typical Culture Collection, Wuhan, China) were cultured in suspension in RPMI 1640 medium (Hyclone, Beijing, China) supplemented with 10% fetal bovine serum (Hyclone), at 37°C and 5%  CO_2_ [10]. Cisplatin (0.5 *μ*g/ml) was added to the COC1/DDP medium to maintain the resistant property; cells were transferred into drug-free medium for >48 h before experiments to avoid interferences due to residual cisplatin [2]. The single-cell suspension was prepared and the concentration was adjusted to 1.0 × 10^6^ cells/ml.

### 2.2. nsEP Exposure

nsEP treatments were performed as described previously using a device designed by School of Physics, University of Electronic Science and Technology of China (Chengdu, China) [4]. 2.0 ml of single-cell suspension was subjected to nsEP. The pulse duration was 32 ns at a 10 Hz pulse repetition frequency, the strength was 10 kV/cm, and the total exposure time was 10 min. nsEP-treated cells were maintained at 37°C before assays. Control cells received sham exposure.

### 2.3. Cell Death

Cell viability was determined with a WST-8 assay (Dojindo Lab., Kumamoto, Japan) after 2, 4, 8, 12, and 24 h, and then the percentage of dead cells was calculated ([1 - (absorbance in treated cells/absorbance in control cells @ 2 h)] × 100%) [11].

### 2.4. DNA Damage

DNA damage was detected with the alkaline comet assay after 2, 4, 8, 12, and 24 h, and cells at 2 and 8 h also received the neutral comet assay to determine whether there was double-strand break (DSB) [12]. The percentage of comet-formed cells was used to quantify the degree of DNA damage [(number of comet-formed cells/number of total cells) × 100%] [13]. Control cells served as the reference considering a high sensitivity of the comet assay: a percentage of <5% demonstrated no unspecific cellular damage, thereby avoiding an overestimation.

### 2.5. Apoptosis

Cell apoptosis was analyzed with the Annexin V assay (Nanjing Keygen Biotech., Nanjing, China) after 2 and 8 h. Cells were stained with FITC-Annexin V and propidium iodide (PI) and then received flow cytometry. The V^+^/PI^−^ population represented early apoptotic cells, the V^+^/PI^+^ population was regarded as late apoptotic cells, and the sum was the number of total apoptotic cells [14].

### 2.6. Mitochondrial Membrane Potential

The membrane potential was determined by fluorospectrophotometry using the JC-1 assay (Invitrogen, Eugene, OR) after 2 and 8 h [15]. *λ*_ex_ was 485 nm, and *λ*_em_ was 529 or 590 nm. The ratio of red to green fluorescence intensity reflected the membrane potential [10, 15]. Cells were also observed under a fluorescence microscopy.

### 2.7. Activity of Caspase-3 and Caspase-9

Activity of caspase-3 and caspase-9 was determined using a luminescent assay (Promega, Madison, WI) after 2 and 8 h. The lg[RLU] (relative light unit) reflected the enzymatic level.

### 2.8. High Mobility Group Box 1 (HMGB1)

HMGB1 in the culture supernatant, the biomarker of cell necrosis, was detected with an enzyme-linked immunosorbent assay (Shino-Test, Kanagawa, Japan) after 2 and 8 h [16].

### 2.9. Temperature Rise

Theoretical simulations suggested that multiple pulses may cause Joule heating [17]. In order to determine whether heat played a part in the cellular response, the temperature rise in the cell suspension was measured using a thermocouple immediately after nsEP exposure (Guangzhou Sungun Meas. Ctrl. Technol. Co., Ltd., Guangzhou, China) [18].

### 2.10. Statistics

Data were processed with the SAS software (SAS Inst., Cary, NC). Analysis of variance was used and multiple comparisons were corrected with the Student-Newman-Keuls test. The critical value was set at p<0.05.

## 3. Results and Discussion

### 3.1. Cell Death and DNA Damage Displayed Two Peak Levels at 2 and 8 h, but Apoptosis Was Detected Only at 8 h

**Figure 1:**
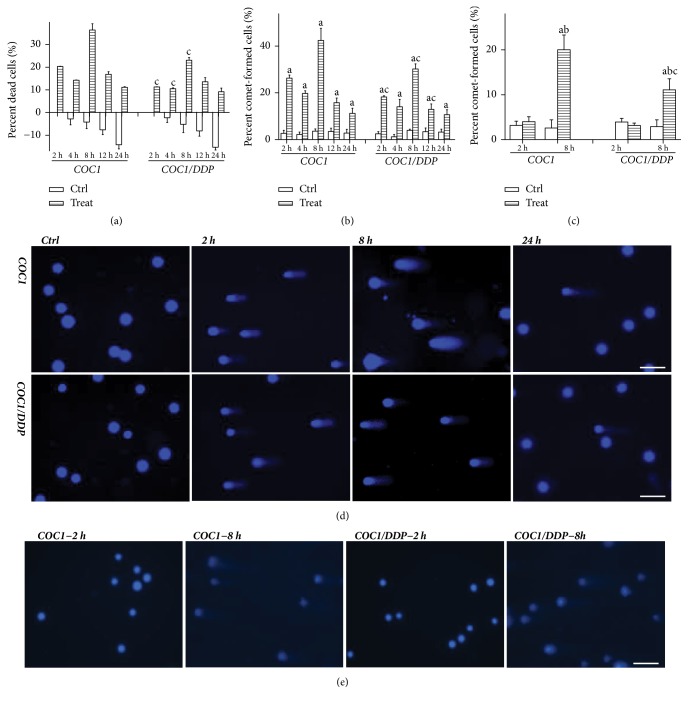
Percentages of dead cells (*a*) and of comet-formed cells in the alkaline assay (*b*) after nsEP treatments: the negative death fraction demonstrated proliferation of control cells; two peak levels were detected at 2 and 8 h, with the highest value at 8 h; the value in COC1 cells was higher than that in COC1/DDP cells. Percentage of comet-formed cells in the neutral assay (*c*): the value was increased at 8 h. Images under the alkaline assay (*d*): more comets were observed at 2 and 8 h in both cell lines; few comets emerged at 24 h, indicating repair. Images under the neutral assay (*e*): comets appeared at 8 h, demonstrating single-strand break at 2 h and double-strand break at 8 h. Data were mean ± standard deviation for 3 independent experiments. The scale bar was 50 *μ*m; (a) versus control, p<0.05; (b) versus 2 h, p<0.05; and (c) versus COC1 at the same time point, p<0.05.

The percentage of dead cells was increased after nsEP treatments in both cell lines, with 2 peak levels noted at 2 and 8 h (p<0.0001, p<0.0001). Values were 20.3±0.1% and 11.3±0.0% at 2 h (p<0.0001), and 36.4±2.7% and 23.1±1.2% at 8 h (p=0.0106), in COC1 and COC1/DDP cells, respectively (Figure 1(a)).

The percentage of comet-formed cells in the alkaline assay displayed a similar trend in both cell lines: the peak value was noted at 2 and 8 h (p<0.0001, p<0.0001). Levels were 26.3±1.3% and 18.2±0.5% at 2 h (p=0.0163), and 42.4±5.2% and 30.2±2.1% at 8 h (p=0.0218), in COC1 and COC1/DDP cells, respectively (Figures 1(b) and 1(d)). These data indicated that nsEP induced early and delayed cellular damage.

In the neutral assay, comets appeared only at 8 h, demonstrating SSB at 2 h and DSB at 8 h. The percentage of comet-formed cells was increased in both cell lines (p=0.0013, p=0.0042), and the value in COC1 cells was higher than that in COC1/DDP cells (20.0±3.3% versus 11.1±2.5%, p=0.0487) (Figures 1(c) and 1(e)).

**Figure 2:**
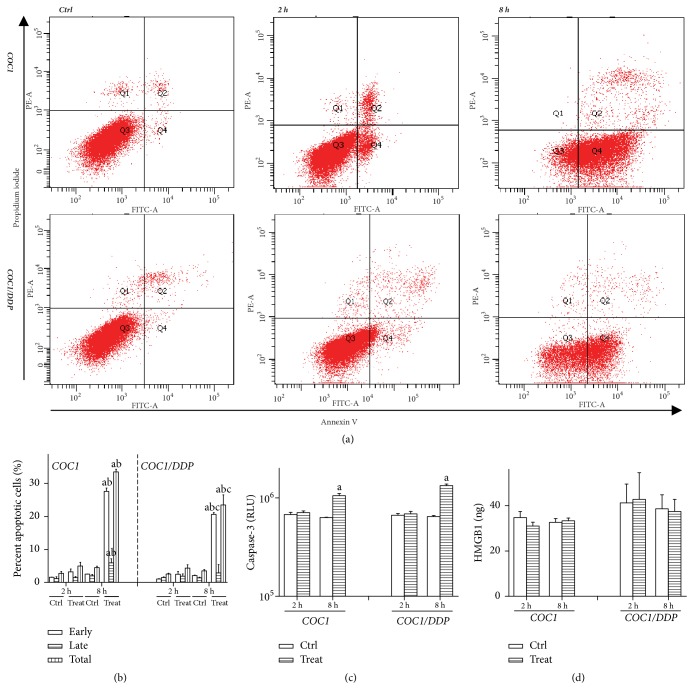
Apoptosis detected with flow cytometry after 2 and 8 h (*a, b*): Q4 represented early apoptotic cells, and Q2 represented late apoptotic cells; a higher apoptotic percentage was detected at 8 h, and the value in COC1 cells was higher than that in COC1/DDP cells. Activity of caspase-3 (*c*): relative light unit (RLU) reflected the enzymatic level; a higher level was noted at 8 h. HMGB1 level (*d*): no increase was detected. Data were mean ± standard deviation for 3 independent experiments. (a) versus control, p<0.05; (b) versus 2 h, p<0.05; (c) versus COC1 at the same time point, p<0.05.

nsEP can directly induce reversibly transient externalization of phosphatidylserine, causing a false positive in identifying apoptotic cells [6, 19]. Therefore, apoptosis was detected ≥2 h after treatments to decrease experimental errors. Apoptosis was analyzed at 2 and 8 h, considering the temporal pattern of cell death and DNA damage. A percentage of <5% demonstrated a lack of apoptosis at 2 h. The apoptotic percentage was increased in both cell lines at 8 h (p<0.0001, p<0.0001), with a higher value in COC1 cells (27.6±1.0% versus 20.6±0.5% for early apoptosis, p=0.0162; 33.5±0.8% versus 23.5±3.1% for total apoptosis, p=0.0355) (Figures 2(a) and 2(b)). The activity of caspase-3 was increased in both cell lines at 8 h (p<0.0001, p<0.0001) (Figure 2(c)). HMGB1 was detected to determine the cell-death mode, since flow cytometry cannot distinguish late apoptotic cells from those membrane-intact necrotic cells [14]. The HMGB1 level was not increased after nsEP exposure (p=0.2594, p=0.4142) (Figure 2(d)). These findings demonstrated that cell death at 8 h was due to apoptosis.

The first peak of cell death and DNA damage occurred 2 h after nsEP exposure. The comet assay showed that DNA damage was SSB. Most SSB can be repaired; certain SSB would evolve into DSB and eventually resulted in cell death via the apoptosis pathway (a programmed process required several hours) [20]. These suggested that cell death at 2 h may not be due to apoptosis. This deduction was supported by alterations of the apoptotic percentage and of caspase-3 activity. Theoretical calculations indicated that the evoked potential in the cellular membrane increased with widening the pulse duration and elevating the strength [4]. Thus, nsEP applied in this study can evoke a potential of about 0.5 V in the cellular membrane (0.2 V was the lowest critical potential required to create membrane pores) [3, 4]. An amount of unrepairable membrane pores would lead to cell lysis [21]. Similar findings were reported in U937 cells: early cell death was detected 1–2 h after nsEP exposure, which resulted from the rupture of plasmic membrane [22]. These results were contrary to the prevalent verdict that nsEP caused repairable nanopores [3, 5, 7, 19]. Previous trials were commonly performed under a single pulse. The present data indicated that membrane pores displayed distinct behaviors under multiple pulses. Continuous pulses can delay the closure of pores and can create new pores, leading to expansion and amalgamation of pores, and ultimately formed large-size pores rupturing the cellular membrane. Pakhomova et al. attributed early cell death to necrosis for lack of caspase-3 activation [22]. Necrosis was a nonprogrammed death mode and was trigged by intracellular damage accompanied with the release of HMGB1 [23]. The HMGB1 level was not increased in the present study. These data demonstrated that early cell death was mainly due to cell lysis. Chemoresistant cells usually had apoptotic deficiency, and the induction of nonapoptotic death can be an alternative therapy [2, 8]. These suggested that nsEP may be a modality against chemoresistant cancer cells.

Romeo et al. observed the DNA electrophoretic pattern in Jurkat cells [24]. DNA migration appeared immediately after single nsEP exposure (60 ns, 25 kV/cm), deteriorated at 20 min, and returned to the baseline level at 1 h; these indicated that nsEP can directly affect the nucleus and that the maximal effect emerged after a certain interval. A strength of 6 kV/cm evoked a potential of 1.98 V in the nucleoplasm [4]. A higher strength with multiple pulses was employed in this study, thereby evoking a longer-lasted higher nucleoplasmic potential (≈3.2 V). Additionally, the temperature rise in the cell suspension was <1°C, demonstrating no involvement of thermal effects. Therefore, SSB at 2 h may result from directly electric effects.

The second peak level of cell death and DNA damage was detected after 8 h. The apoptotic percentage was increased and approached to the cell-death percentage; caspase-3 was activated, and there was no increase of the HMGB1 level. The time span from SSB to apoptosis commonly was several hours [20]. A long interval between nsEP treatments and the activation of caspase-3 was consistent with the theoretical simulations of Song et al., where the caspase-3 level elevated after 5 h in mitochondrial apoptosis due to nsEP [25]. These data manifested that cell death at 8 h was due to apoptosis.

### 3.2. Mitochondrial Damage Was Observed at 8 h

**Figure 3:**
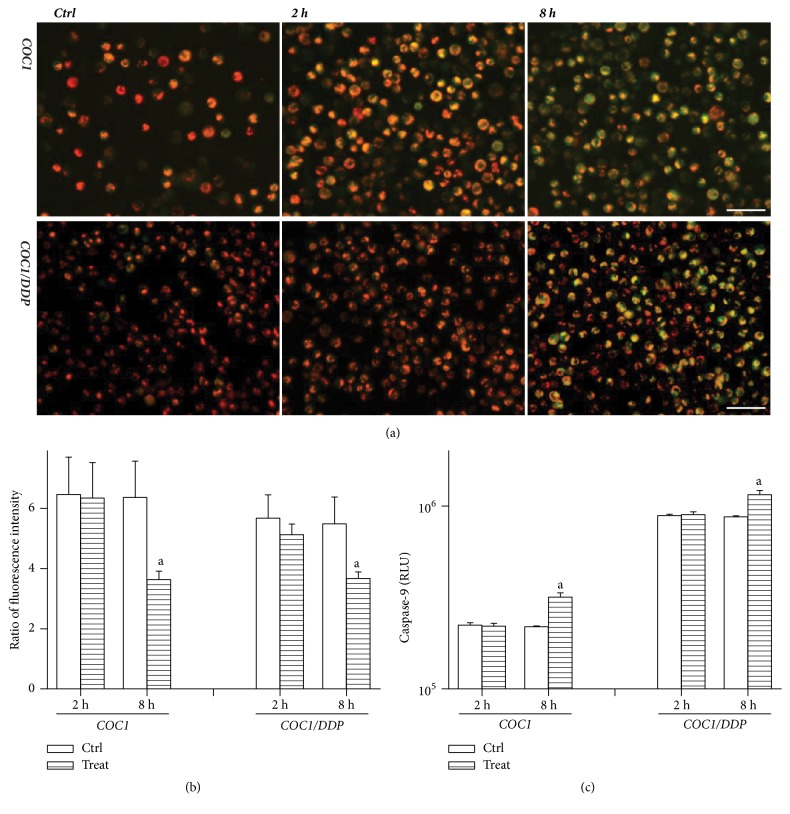
Mitochondrial membrane potential detected with the JC-1 assay. Images under fluorescence microscopy (*a*): green fluorescence was due to the monomer of JC-1 and the number of green cells was increased at 8 h, indicating collapse of the potential. The membrane potential qualified with the ratio of fluorescence intensity (*b*): the potential was decreased at 8 h. Activity of caspase-9 at 2 and 8 h (*c*): relative light unit (RLU) reflected the enzymatic level; the enzymatic activation was detected at 8 h. Data were mean ± standard deviation for 3 independent experiments. The scale bar was 50 *μ*m; (a) versus control, p<0.05.

The JC-1 ratio was decreased at 8 h and no variation was observed at 2 h, in both COC1 and COC1/DDP cells (p=0.0074, p=0.0158) (Figures 3(a) and 3(b)). The level of activated caspase-9 was increased in both cell lines at 8 h (p<0.0001, p<0.0001) (Figure 3(c)). These data demonstrated the occurrence of mitochondrial damage.

A strength of 6 kV/cm can evoke a potential of 1.17 V in the nucleoplasm [4]. Thus, nsEP applied in this study can evoke a higher cytoplasmic potential (≈1.9 V) to impair mitochondria, initiating apoptosis [3, 26]. The collapse of mitochondrial membrane potential and activation of caspase-9 demonstrated mitochondrial insults and mitochondria-dependent apoptosis. This result accorded with the prevalent verdict that nsEP deactivated cells via apoptosis [6, 7, 19]. A translocation of apoptosis inducing factor from mitochondria into the nucleus led to cleavage of DNA, thereby resulting in DSB [27]. Because DNA break mainly resulted from apoptosis, the gap between the percentage of comet-formed cells and that of dead/apoptotic cells was relatively narrow.

### 3.3. Potential Therapeutic Applications

These two phases of cell death had distinctly therapeutic implications. The first phase was mainly due to cell lysis, which can be used to necrotize cancer tissues directly. The second phase was due to apoptosis, thereby being a strategy to enhance the action of an apoptotic therapy (e.g., chemotherapy). Therefore, nsEP can deactivate resistant cells via multiple pathways. This was an advantage since chemoresistance resulted from many overlapped mechanisms—the induction of a sequence of cell death can lead to synergism to improve the therapeutic efficacy. The temporal pattern of cell death was a reference to set the interval for administrating other treatments [21].

A less percentage of dead/comet-formed cells after 8 h demonstrated the cellular repair. A similar result was observed in insonated cells: the proliferation capacity was improved in certain subpopulations causing compensational effects, which was related to the heterogeneity of cancer cells [28–30]. Cells with a higher repair capacity had a higher compensation capacity. This may play a part in the lower toxicity noted in COC1/DDP cells and should be explored to formulate a protocol to set the interval between therapeutic courses.

The evoked potential in a subcellular unit was determined by nsEP applied (strength and the pulse duration), electric property of contents (conductivity and permittivity), and absolute and relative sizes of a cell [19]. Thus, for a specific cancer type, an expected potential within cancer cells can be realized by modulating the strength and pulse duration, improving the therapeutic effect. nsEP caused lower cytotoxicity in chemoresistant cells; underlying mechanisms should be explored in follow trials.

## 4. Conclusion

nsEP induced an early phase of cell death and caused SSB. nsEP induced apoptosis leading to a late phase of cell death, which related to mitochondria insults. The temporal pattern of DNA break and cell death may have distinct implications to treatments of resistant ovarian cancers.

## Data Availability

The data used to support the findings of this study are included within the article.
